# Healthcare costs of different treatment options for condylar fractures

**DOI:** 10.1016/j.heliyon.2023.e19851

**Published:** 2023-09-09

**Authors:** Loreine M.L. Helmer, Leander Dubois, Frank Lobbezoo, Jan de Lange, Judith E. Bosmans

**Affiliations:** a Department of Oral and Maxillofacial Surgery, Academic Medical Centre of Amsterdam (AUMC) and Department of Orofacial Pain and Dysfunction, Academic Centre for Dentistry (ACTA), University of Amsterdam and Vrije Universiteit Amsterdam, Meibergdreef 9, AZ Amsterdam ZO, Amsterdam, 1105, the Netherlands; b Department of Oral and Maxillofacial Surgery, Academic Medical Centre of Amsterdam (AUMC), Academic Centre for Dentistry (ACTA), University of Amsterdam, Amsterdam, Meibergdreef 9, AZ Amsterdam ZO, 1105, the Netherlands; c Departments of Oral Health Sciences and of Orofacial Pain and Dysfunction, Academic Centre for Dentistry (ACTA), University of Amsterdam and Vrije Universiteit Amsterdam, Amsterdam, Gustav Mahlerlaan 3004, 1081, LA, Amsterdam, the Netherlands; d Department of Oral and Maxillofacial Surgery, Academic Medical Centre of Amsterdam (AUMC), Academic Centre for Dentistry (ACTA), University of Amsterdam, Meibergdreef 9, AZ Amsterdam ZO, Amsterdam, 1105, the Netherlands; e Section Health Technology Assessment, Department of Health Sciences, Faculty of Science, Vrije Universiteit Amsterdam, Amsterdam Public Health Research Institute, Van der Boechorststraat 7, 1081, BT Amsterdam, the Netherlands

## Abstract

**Objective:**

As treatment options for condylar fractures have comparable outcomes, getting insight into associated costs is a first step towards implementing value-based healthcare (VBH). Therefore, we described the actual costs of the different treatment options (surgical, conservative, and expectative treatment) for condylar fractures. We expected surgical treatment to be the most expensive treatment.

**Study design:**

This is a cost-of-illness study, based on estimates from the literature. Firstly, care pathways of all treatment options were described. Secondly, the costs per step were calculated based on the literature and Dutch guidelines for economic evaluations in health care.

**Results:**

The direct treatment costs of surgical treatment (€3721 to €4040) are three to five times higher than conservative treatment (€730 to €1332). When lost productivity costs during the recovery period are included, costs of surgical treatment remain 1.5 times higher (€9511 to €9830 for surgical treatment and €6224 to €6826 for conservative treatment). The costs of expectative treatment (€5436) are lower than both other treatments.

**Conclusion:**

The costs for surgical treatment are considerably higher than those for conservative or expectative treatment, mainly related to direct treatment cost. Future research should focus on the patients’ perspective, to support implementation of VBH in treating condylar fractures.

## 1 Introduction

In the Netherlands, there is no literature on the incidence and prevalence of condylar fractures. However, based on data of three large Dutch Oral and Maxillofacial Surgery departments, it was estimated that around 500 patients sustain a condylar fracture each year. Even though this is a relatively small number of patients, care for patients with condylar fractures could have a substantial budgetary impact. However, it is unclear whether actual costs are considerably different between different treatment options for patients with condylar fractures. If treatment options vary broadly in costs, taking those costs into account when choosing between treatment options could reduce this budgetary impact.

In the case of a condylar fracture, a maxillofacial surgeon has two main treatment options: surgical or conservative treatment. Surgical treatment includes open fracture reduction and fixation with osteosynthesis material. Conservative treatment consists of securing the occlusion with arch bars, intermaxillary fixation screws (IMF screws) or brackets combined with guiding elastics. Conservative treatment sometimes also consists of expectative care, which means that no treatment is provided, but the patient is followed during the healing process. While surgical treatment focuses on restoring the anatomy, conservative treatment focuses on restoring occlusion and function. Several clinical trials have tried to compare surgical and conservative treatment options. However, they have faced various obstacles, ranging from small numbers of people willing to participate to difficulties setting up and maintaining a uniform study protocol to be used in the different participating centres [1]. Reviews comparing surgical and conservative treatment are also hampered by the lack of uniformity in treatment protocols [[2], [3], [4], [5]]. Nonetheless, these reviews show that both treatment options yield promising results in terms of jaw function and low failure rates [3,4,6,7].

Condylar fractures can be anatomically divided into three categories, namely those involving the head, neck, and base of the condyle [8]. Thereby, the condylar head reference line and the sigmoid notch line divide the three structures. Although location of condylar fracture might influence the treatment of choice, fractures in all three areas can be treated surgically and conservative [8].

When there is no consensus on the optimal treatment for a specific health condition with regard to treatment outcomes, as is the case for condylar fractures [2,3], other arguments need to be used for decision making. The value-based healthcare (VBH) framework can support healthcare providers in making these choices, but has not yet been introduced widely in the field of oral and maxillofacial surgery (OMFS). To be able to apply the concept of VBH in the care of patients with condylar fractures, it is necessary to get insight into the actual costs of the different treatment options, next to the effects on the patient. To our knowledge, these costs have never been compared before. We aimed to estimate the direct treatment and follow-up costs of the different treatment options for condylar fractures.

## 2 Materials and methods

This cost-of-illness study described the costs of the three treatment options for condylar fractures – surgical, conservative, and expectative treatment. All healthcare utilization and cost estimates were based on recommendations in the Dutch clinical guidelines for condylar fractures and the literature. If necessary, expert opinion was used.

**Fig. 1:**
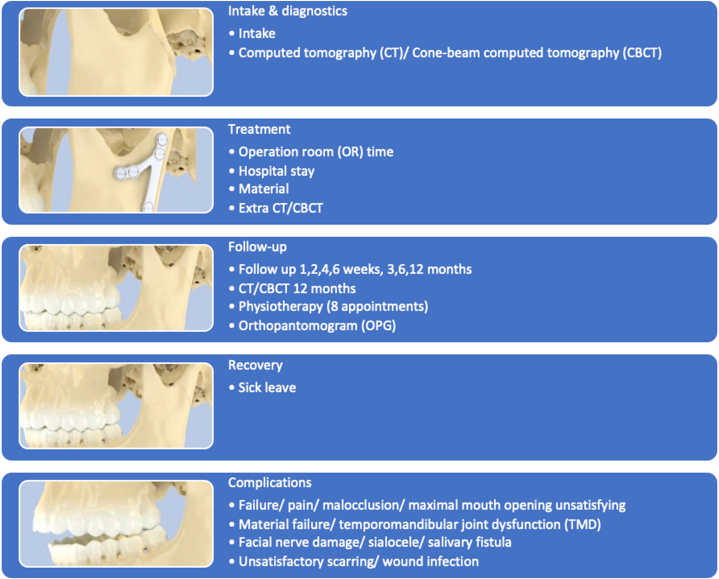
Care pathway for surgical treatment: *all phases of the care pathway the patient could pass after condylar fractures and surgical treatment*.

**Fig. 2:**
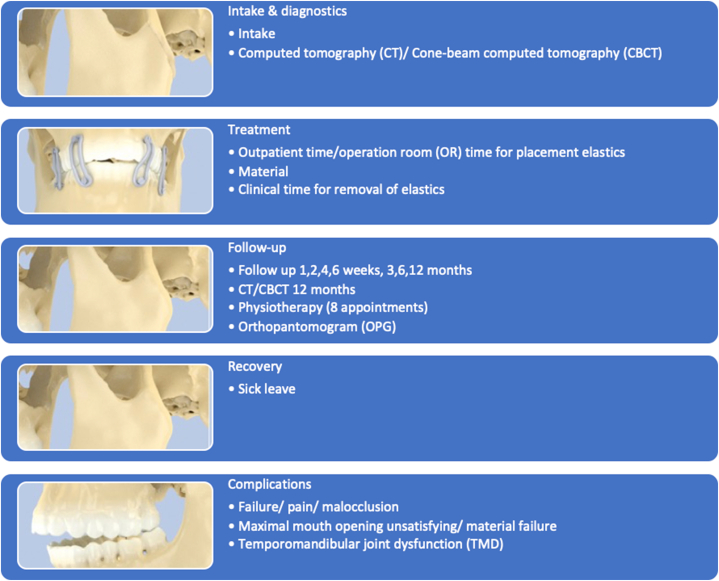
Care pathway for conservative treatment: all phases of the care pathway the patient could pass after condylar fractures and conservative treatment.

**Fig. 3:**
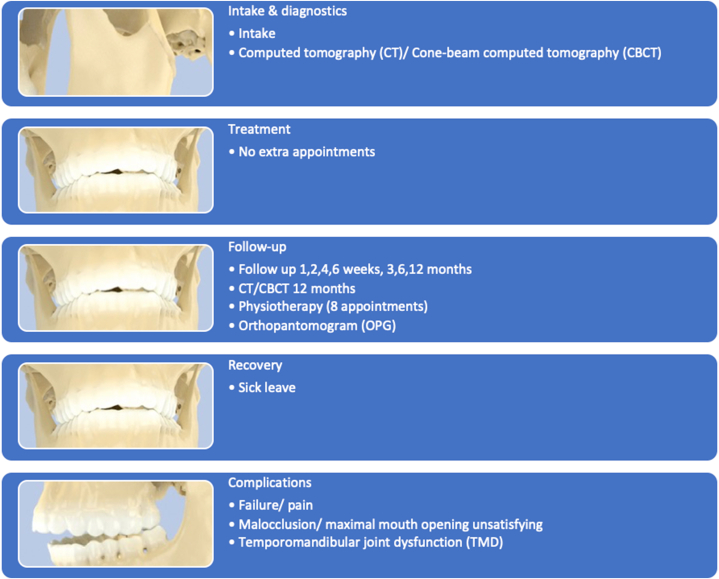
Care pathway for expectative treatment: *all phases of the care pathway the patient could pass after condylar fractures and expectative treatment*.

A bottom-up approach was used to collect the necessary information on the three treatment options and their associated costs. First, the care pathways for each of the three different treatment options were described containing all elements of the treatment, starting with intake and diagnostics, followed by the treatment itself and the follow-up appointments. The care path also includes the recovery and a description of the possible complications related to the treatment (see Fig. 1, Fig. 2, Fig. 3). The care pathways were based on the usual care provided in four different Dutch hospitals, viz., two university hospitals and two general hospitals. The care pathways represent a general overview of the care most patients receive; this may vary, however, as all patients and fractures are different. For example, less severe cases might not need extensive follow-up. Secondly, the costs associated with each element in the care pathways were estimated. Finally, the complications per treatment were described and costed separately.

### 2.1 Costs

Costs were divided into direct treatment costs, i.e., the costs for the intake and diagnostics and the treatment itself, and follow-up costs, and indirectly related costs, i.e., costs related to recovery and complications. Only the costs of condylar fractures were investigated. Extra costs for concomitant fractures were not considered, because treatment of concomitant fractures may weigh heavily on the costs and could mask the difference between costs of the different treatment options for condylar fractures.

Resource utilization associated with each of the three options was valued, based on the recommendations in the Dutch guideline for conducting economic evaluations in healthcare [9]. For all hospital-specific costs, an average of the costs of academic and non-academic medical centres was used. The costs of materials were estimated based on the prices of a company that provides materials for both surgical and conservative treatment. Other costs, such as an operation room per hour, were based on literature. As different material options are available for surgical treatment and conservative treatment, the costs were not easily covered by one number. Instead, a best- and worst-case scenario per treatment was calculated, taking different materials and application time per material into account.

Besides the costs directly related to the treatment and follow-up of condylar fractures, there may also be costs associated with sick leave during or after treatment that contribute to the total costs. Patients may be absent from work during recovery due to a hospital stay, pain, or other complaints. A more extended recovery period will lead to more costs due to sick leave. The number of sick leave days per treatment was based on three different scenarios, as the literature on this subject was insufficient. The costs per day of sick leave were also obtained from the Dutch guideline for conducting economic evaluations in healthcare [9]. The type of complications and associated costs vary per treatment option and can significantly affect the costs of the different treatment options. The risk of the complication occurring was extracted from the literature for each complication separately. Based on the sample size per study, a percentage of the risk was calculated. The costs for each complication were weighed against the incidence of the complication to calculate the costs per patient for each complication.

## 3 Results

Table 1 shows the distribution of costs per treatment option and the total costs of the treatment options. Costs of human resources mainly contain the time and, thus, salary costs of healthcare providers. Diagnosis and material costs include computed tomography (CT), cone-beam computed tomography (CBCT) scans, orthopantomograms (OPG) and the platelets or brackets. For all three treatment options, personnel costs contributed most to the total direct treatment costs of the treatment option. A detailed overview of all different costs elements is presented in Table S1 of the supplementary information. The lowest costs were those of the expectative treatment, which amounted to €5436 from intake until the end of treatment including costs of recovery and possible complications. The costs of conservative treatment varied from €6224 to €6826. The surgical treatment was associated with the highest costs, which were estimated to be between €9511 and €9830. An overview of the different intake and diagnostics and follow-up components can be found in Supplementary Tables S1 and S2.

**Table 1:** Summary of the costs for treatment of condylar fractures.

Overview Costs	Surgical treatment	Conservative treatment	Expectative treatment
Costs intake & diagnostics	388.0	388.0	388.0
Human resources	259.0	259.0	259.0
Materials & diagnostics^∗^	129.0	129.0	129.0
**Costs treatment best**	**3245.0**	**931.0**	**0.0**
Human resources	2429.5	273.0	
Materials & diagnostics^∗^	550.0	112.0	
**Costs treatment worst**	**4381.3**	**853.0**	**0.0**
Human resources	2922.0	136.5	
Materials & diagnostics^∗^	265.0	580.0	
**Costs follow-up**	**1080.0**	**1080.0**	**1080.0**
Human resources	901.0	901.0	901.0
Materials & diagnostics^∗^	179.0	179.0	179.0
**Costs recovery**	**3892.0**	**3892.0**	**3892.0**
Human resources	1946.0	5838.0	5838.0
Materials & diagnostics^∗^	0.0	0.0	0.0
**Costs complications**^**∗∗∗**^	**416.7**	**188.6**	**129.4**
Human resources	338.9	159.6	112.5
Materials & diagnostics^∗^	77.8	28.9	16.9
**Total costs best**	**9021.7**	**6479.6**	**5489.4**
**Total costs worst**	**10158.0**	**6401.6**	**5489.4**

All costs in euro's *costs materials and diagnostics include computed tomography (CT), cone-beam computed tomography (CBCT), orthopantomogram (OPT), screws, platelets.

**because of different material options for surgical and conservative treatment, this value is a range.

***costs of complication is a combination of all possible extra costs per complication multiplied by the percentage of their occurrence.

### 3.1 Treatment cost

The difference in costs between the best- and worst-case scenarios for surgical and conservative treatment can be explained by the use of different materials; some osteosynthesis systems are more expensive than others or take more time to apply. Table 2 shows that surgical or conservative treatment materials can substantially differ in costs. For example, in surgery with arch bars, the costs of operation room (OR) time outweigh the more expensive but faster treatment option with materials such as IMF screws or even hybrid maxillomandibular fixation (MMF). For conservative treatment, the opposite is true; the use of IMF screws or brackets is far less expensive than other materials because of the rent and, therefore, the time to apply the brackets is far less expensive in an outpatient setting compared to the OR. Overall, the difference in costs between surgical and conservative treatment is substantial; surgical treatment costs outweigh conservative treatment by a factor of thee or in worst case scenarios even a factor five, which is mainly explained by the difference in costs between inpatient and outpatient time and the additional hospital stay after surgical treatment.

**Table 2:** Specification of the costs for different treatments of condylar fractures, including various types of material, without further checkups.

Care pathway	Details	Costs minimal	Costs maximal	Source
**Costs surgical treatment**				
*Human resources:*				
Operation room (OR) time (including disposables), 1 h		985		Helkio et al. (2016) [19], Raft et al. (2015) [20], Childers and Maggard-Gibbons (2018) [21], Patel et al. (2020) [22]
Hospital stay 2 nights	Patient day, weighted average different type hospital, €476	952		Richtlijn Zorginstituut [9]
Clinical time 15min (including disposables)	Clinical appointment, weighted average, 91 €/10 min	136.5		Richtlijn Zorginstituut [9]
*Materials & diagnostics*				
Osteosynthesis material (sub-condylar plate, 4 screws 2.0, drill bit)	Subcondylar plate four holes €75, 2.0 screw €20, drill bit (outside NL single use) €75	230		Strykerr^∗∗∗∗^
Arch Bars	Arch bars €35	35		Edmunds et al. (2019) [23]
Intermaxillary fixation (IMF) screw (4–6 screws per side)	Screw €40	320	480	Strykerr^∗∗∗∗^
Hybrid maxillomandibular fixation (MMF) material (2 plates and 8 screws)	Plate €150, screw €35	580		Strykerr^∗∗∗∗^
Computed tomography (CT) or Cone-beam computed tomography (CBCT), brain image		129		Richtlijn Zorginstituut [9]
**Costs surgical treatment with arch bars**	2 h and 15min OR time, hospital stay, osteosynthesis material & arch bars, removal outpatient clinic & dental hygienist 1.5 h	**4381.25**		
*Human resources*		3741		
*Materials & diagnostics*		394		
**Costs surgical treatment with IMF screws**	1.5 h OR time, hospital stay, osteosynthesis material & IMF screws, removal outpatient clinic 15 min	**3245**	3139.5	
*Human resources*		2566		
*Materials & diagnostics*		679	710	
**Costs surgical treatment with hybrid MMF material**	1.5 h OR time, hospital stay, osteosynthesis material & hybrid MMF, removal outpatient clinic 15 min	**3505**		
*Human resources*		2566		
*Materials & diagnostics*		939		
				
***Total costs surgical treatment, best***	Surgical treatment with IMF screws	**3245**		
*Human resources*		2429.5		
*Materials & diagnostics*		550		
***Total costs surgical treatment, worst***	Surgical treatment with arch bars	**4381.25**		
*Human resources*		2922		
*Materials & diagnostics*		265		
**Costs conservative treatment**				
*Human resources:*				
Clinical time 15min (including disposables)	Clinical appointment, weighted average, 91 €/10 min	136.5		Richtlijn Zorginstituut [9]
*Materials & diagnostics*				
Arch Bars	Arch bars €35	35		Edmunds et al. (2019) [23]
Brackets	Brackets 32 euro per 8 teeth > for 28 teeth >32^∗^3.5 = 112 euro	112		Wiedel et al. (2016) [24]
IMF screw (4–6 screws per side)	Screw €40	320	480	Strykerr^∗∗∗∗^
Hybrid MMF material (2 plates and 8 screws)	Plate €150, screw €35	580		Stryker^∗∗∗∗^
**Costs conservative treatment with arch bars**	1 h clinical time, arch bars, removal outpatient clinic & dental hygienist 1.5 h	**1400**		
*Human resources*		1365		
*Materials & diagnostics*		35		
**Costs conservative treatment with brackets**	30 min clinical time, brackets, removal outpatient clinic & dental hygienist 1 h	**931**		
*Human resources*		819		
*Materials & diagnostics*		112		
**Costs conservative treatment with IMF screws**	15 min clinical time, IMF screws & removal outpatient clinic 15 min	**593**		
*Human resources*		273		
*Materials & diagnostics*		320		
**Costs conservative treatment with hybrid MMF material**	15 min clinical time, hybrid MMF material & removal outpatient clinic 15 min	**853**		
*Human resources*		273		
*Materials & diagnostics*		580		
***Total costs conservative treatment, best***	Conservative treatment with brackets	**931**		
*Human resources*		273		
*Materials & diagnostics*		112		
***Total costs conservative treatment, worst***	Conservative treatment with hybrid MMF materials	**853**		
*Human resources*		136.5		
*Materials & diagnostics*		580		

All costs in euro's *Costs materials and diagnostics include computed tomography (CT), cone-beam computed tomography (CBCT), orthopantomogram (OPT), screws, platelets.

**because of different material options for conservative treatment, this value is a range.

***costs of complication is a combination of all possible extra costs per complication multiplied by the percentage of their occurrence.

****Stryker, company for medical technology.

### 3.2 Recovery costs

**Fig. 4:**
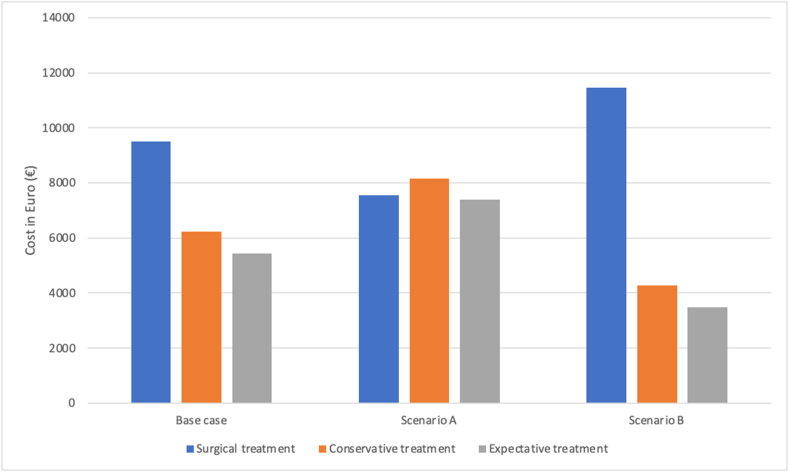
Total costs per treatment for three different recovery scenarios. Three different scenarios of the recovery period after treatment of a condylar fracture. For all treatment options, the less expensive, ‘best’ treatment option was used.Our study uses a base case with 14 days of sick leave for every treatment option. Scenario A includes seven days of sick leave after surgical treatment and 21 days of sick leave after conservative and expectative treatment. Scenario B includes 21 days of sick leave after surgical treatment and seven days of sick leave after conservative and expectative treatment.

For all treatment options, sick leave during the recovery period was one of the largest contributors to total costs for all treatment options. Due to the fact that there is only scarce literature available on the number of sick leave days after condylar fracture, three different scenarios are presented in Fig. 4. The details of Fig. 4 are represented in Supplementary Table S3. We assumed 14 days of sick leave for all different treatment options in the base case analysis. In scenario A, sick leave after surgical therapy is shortened to 7 days, whereas sick leave after conservative and expectative treatment is extended to 21 days. In scenario B, sick leave after conservative and expectative treatment is shortened to 7 days, and sick leave after surgical treatment is extended to 21 days. These different assumptions have an enormous impact on the total costs for the different treatments, as shown in Fig. 4. In case a recovery period for conservative treatment is at least 12 days longer than the recovery period for surgical treatment, conservative treatment is the most expensive of both. For expectative treatment, this difference must be 15 days or longer, in order for the total sum of expectative treatment to be more expensive than surgical treatment.

### 3.3 Complication cost

Table 3 summarizes the prevalence of possible complications after treatment of condylar fractures. Costs of complications contributed about 5–10% to the total costs of the different treatments. Although possible complications varied per treatment, treatment failure and pain occurred with each treatment option. Compared to the other treatment options, surgery resulted in more diverse complications, including plate failure, a sialocele (a cyst within the salivary gland), and unsatisfactory scarring, which may lead to extra costs. As a result, the costs for surgical complications were more than three times higher than for the other treatment options.

**Table 3:** Complication overview per treatment.

Care pathway	Treatment	Occurrence in percentage	Costs (€)	Cost ()/percentage	Source
**Costs complications surgical treatment**					
Failure treatment	Extra operation^a^	0.05	3245.0	158.8	Richtlijn Zorginstituut [9], Stryker^b^, Helkio et al. (2016) [19], Raft et al. (2015) [20], Childers and Maggard-Gibbons (2018) [21], Patel et al. (2020) [22]
Pain	Medication (Ibuprofen 14 days, 0.56 €/day)	0.08	7.8	0.6	Website medical costs [25]
Malocclusion	Clinical appointment, lowering dental elements	0.04	136.5	6.1	Richtlijn Zorginstituut [9]
Maximal mouth opening unsatisfying	8 extra physiotherapy sessions	0.06	264.0	17.1	Richtlijn Zorginstituut [9]
Material failure (plate fracture, torsion, screw loosening)	Extra operation, new plate/screw^a^	0.05	2487.0	118.8	Richtlijn Zorginstituut [9], Stryker^b^, Helkio et al. (2016) [19], Raft et al. (2015) [20], Childers and Maggard-Gibbons (2018) [21], Patel et al. (2020) [22]
Facial nerve damage (temporary & permanent)	8 mime training sessions	0.11	272.0	29.2	Richtlijn Zorginstituut [9]
Sialocele	Operation removal sialocele, extra computed tomography (CT) or cone-beam computed tomography (CBCT), brain image, medication (Rovinol 7 days, 12.25 €/day)	0.02	1199.8	25.0	Website medical costs [25], Richtlijn Zorginstituut [9], Stryker^b^, Helkio et al. (2016) [19], Raft et al. (2015) [20], Childers and Maggard-Gibbons (2018) [21], Patel et al. (2020) [22]
Salivary fistula	Appointment at clinic, CT/CBCT (brain image), abces drainage, medication (Rovinol 7 days, 12.25 €/day)	0.04	351.3	14.5	Website medical costs [25], Richtlijn Zorginstituut [9]
Unsatisfactory scarring	Operation to improve scar	0.00	985.0	2.8	Richtlijn Zorginstituut [9], Stryker^b^, Helkio et al. (2016) [19], Raft et al. (2015) [20], Childers and Maggard-Gibbons (2018) [21], Patel et al. (2020) [22]
Wound infection	Appointment at clinic, extra CT/CBCT (brain image), abces drainage	0.03	265.5	7.5	Richtlijn Zorginstituut [9]
Temporomandibular joint dysfunction (TMD)	8 extra physiotherapy sessions, an appointment at clinic and stabilization splint (night guard)	0.07	496.5	36.3	Richtlijn Zorginstituut [9] &Elysee Dental^c^
***Total costs complications surgical treatment***				**416.7**	
***Human resources***				**338.9**	
***Materials & diagnostics***				**77.8**	
**Costs complications conservative treatment**					
Failure treatment	Extra operation^a^	0.00	3245.0	6.1	Richtlijn Zorginstituut [9], Stryker^b^, Helkio et al. (2016) [19], Raft et al. (2015) [20], Childers and Maggard-Gibbons (2018) [21], Patel et al. (2020) [22]
Pain	Medication (Ibuprofen 14 days, 0.56 €/day)	0.09	7.8	0.7	Website medical costs
Malocclusion	Clinical appointment, lowering dental elements	0.12	136.5	17.0	Richtlijn Zorginstituut [9]
Maximal mouth opening unsatisfying	8 extra physiotherapy sessions	0.10	264.0	27.3	Richtlijn Zorginstituut [9]
Material failure (fracture/loosening brackets/intermaxillary fixation (IMF)/maxillomandibular fixation (MMF))	Extra Clinical appointment, new fracture/bracket/screw^a^	0.35	171.5	59.2	Richtlijn Zorginstituut [9], Stryker^b^
Temporomandibular joint dysfunction (TMD)	8 extra physiotherapy sessions, an appointment at clinic and stabilization splint (night guard)	0.16	496.5	78.4	Richtlijn Zorginstituut [9] &Elysee Dental^c^
***Total costs complications conservative treatment***				**188.6**	
***Human resources***				**159.6**	
***Materials & diagnostics***				**28.9**	
**Costs complications expectative treatment**					
Failure treatment	Extra operation^a^	0.00	3245.0	6.1	Richtlijn Zorginstituut [9], Stryker^b^, Helkio et al. (2016) [19], Raft et al. (2015) [20], Childers and Maggard-Gibbons (2018) [21], Patel et al. (2020) [22]
Pain	Medication (Ibuprofen 14 days, 0.56 €/day)	0.09	7.8	0.7	Website medical costs [25]
Malocclusion	Clinical appointment, lowering dental elements	0.12	136.5	17.0	Richtlijn Zorginstituut [9]
Maximal mouth opening unsatisfying	8 extra physiotherapy sessions	0.10	264.0	27.3	Richtlijn Zorginstituut [9]
Temporomandibular joint dysfunction (TMD)	8 extra physiotherapy sessions, an appointment at clinic and stabilization splint (night guard)	0.16	496.5	78.4	Richtlijn Zorginstituut [9] &Elysee Dental^c^
***Total costs complications expectative treatment***				**129.4**	
***Human resources***				**112.5**	
***Materials & diagnostics***				**16.9**	

a In case of retreatment, the less expensive/'best’ option was elected.

b Stryker, company for medical technology.

c Elysee Dental, company for dental technology.

## 4 Discussion

The results show that there is a considerable difference in costs between surgical, conservative, and expectative treatment of condylar fractures. The total costs of the surgical care pathway is around 1.5 times higher than those of conservative and expectative care. This can be mainly explained by the difference in the direct costs of the treatment itself, which for surgical treatment outweighs conservative treatment by a factor between three to five. If IMF screws or even hybrid MMF are used during surgery for condylar fractures, OR costs for treating inpatients will be lower despite the use of more expensive materials due to time savings. The opposite is true for outpatient treatments, which are less expensive because there is no need for hiring an OR. So, in these cases using the less expensive IMF or brackets is more cost-effective. This does not apply to all materials however; in case of arch bars the time of application and corresponding costs are too high to be compensated by the less expensive material. Application of arch bars are therefore the most expensive option in surgical treatment, as well as conservative treatment. Complications represent only a minor part of the total costs. These costs differed substantially per treatment, with costs of post-surgical complications being almost three times higher than those after conservative treatment. Although recovery is an essential part of the care pathway that weighs heavily on the total cost, there is little information on the length of the recovery period. Therefore, we assumed that all patients were absent from paid work for 14 days, regardless of the type of treatment.

While we only focused on condylar fractures, trauma sometimes causes concomitant fractures. Only 82 out of 368 patients in a study by Zachariades et al. (2006) were diagnosed with a solitary unilateral condylar fracture. Twenty-one had bilateral condylar fractures, and 265 had a concomitant orofacial fracture, often a second mandibular fracture [10]. These patients always need surgical treatment for secondary fractures. As the hospital stay (at a cost of almost €500 a day) in these cases would already be necessary for the other fractures, the costs of treating the primary – condylar – fracture surgically would not include these costs for the hospital stay. However, even in this scenario the costs of the surgical treatment would remain the highest of all treatments.

As with bilateral fractures, we did not take the severity of the condylar fracture into account. This study is based upon the principle of comparability of fracture and equal treatment options. If there would be cases in which one of both treatment options is favoured above the other, a cost-comparison of treatment options has no value. So this cost-of-illness study has value only in cases were both treatment options have comparable clinical outcomes, as shown in reviews on the subject [2,3].

The total costs of a care pathway is also affected by the costs of the follow-up care consisting of physiotherapy, which we considered to be identical for surgical and conservative treatment. Although there is no consensus in the literature on the number of physiotherapy sessions needed after surgical or conservative treatment, maxillofacial surgeons might argue that more physiotherapy is necessary after conservative treatment than after surgical treatment. Despite literature showing that 6% of patients needed extra physiotherapy after surgery whereas 10% needed extra physiotherapy after conservative treatment [3,6,7], it is not entirely clear whether patients received more physiotherapy sessions after conservative treatment compared to surgical treatment. On the other hand, if the costs of physiotherapy are doubled, the costs of conservative treatment would rise by only 10%. Thus, even in this scenario, the conservative treatment would be by far the least expensive option.

As stated earlier, information on the recovery period after a condylar fracture is limited. Although Lindqvist's study found 14 days of sick leave after mandibular fractures, this was a mean for all different treatment options, and no specific figures were provided for condylar fractures alone [11]. Another study on the surgical treatment of mandibular fractures used eight to ten days of sick leave [12], but, unfortunately, no data sources for this number were provided, and condylar fractures were not mentioned in this study. Van den Bergh found that patients with condylar fractures had an average of 20 days of sick leave after conservative treatment [13]. However, these results are not comparable, since the majority of the patient population included by Van den Bergh et al. suffered concomitant mandibular fractures (86%), and all were treated in the operating room. So, patients had to recuperate from more than just a unilateral condylar fracture and also from the general anaesthetic. While one of the great advantages of conservative treatment is the possibility to avoid a general anaesthetic and treat a patient in an outpatient clinic. We expected that treatment in an outpatient setting would lead to fewer days of sick leave compared to an operation under general anaesthetic. However, the different scenarios on sick leave (Fig. 4) show that only if the outpatient treatments lead to a longer recovery period of 12 days for conservative and 15 days for expectative treatment, the surgical treatment will be the least expensive option.

In the present study, we only looked at costs, whereas treatment outcomes are also important. Most studies evaluating outcomes of treatments for condylar fractures focussed on objective outcomes only, such as the range of motion and millimetres of mouth opening. These objective outcomes might be of less interest to patients, whereas outcomes such as pain and recovery period may be more relevant in the perception of patients. However, such aspects have not been described yet [14]. Moreover, although the literature shows that mandibular fractures and their treatments can negatively affect oral health related-quality of life (OHRQoL) [15,16], there are very few studies describing OHRQoL after treatment for condylar fractures [16]. Omeje et al. concluded that there was no difference in OHRQoL between different treatment options for condylar fractures [16]. Contrary to this result, Naik found that patients experienced less subjective pain and better function after surgical treatment compared to conservative treatment [17]. However, Naik et al. caution that possibly the more severe complications of surgical treatment were missed due to the small sample size [17]. More studies on OHRQoL, primarily focussing on pain and complications of treatment, but also on recovery and days of sick leave, are important to be able to fully describe the burden of condylar fractures for patients and society.

Value-based healthcare (VBH) is concerned with achieving the best possible outcomes for patients at the lowest possible costs [18]. Therefore, besides the cost information provided by the current study, information on patient outcomes is crucial to be able to integrate value-based healthcare in the care for patients with condylar fractures in the future. Our study takes the first steps towards the implementation of VBH in the treatment of condylar fractures by describing the care pathways belonging to the different treatment options and giving an elaborated overview of costs associated with these care pathways. However, our study also shows that there are some additional questions that need answering. The most important questions are whether there are differences in recovery between the different treatment options, whether recovery time and possible complications of treatment are important factors from patients’ perspective, whether there is a difference in OHRQoL between different types of treatment for a condylar fracture, and whether this difference is retained after a follow-up period. More research to answer these specific questions will be necessary to fully implement VBH in the care for condylar fractures in the future. A future study on patients with condylar fractures, with particular attention to their OHRQoL and their recovery, would provide the missing information.

The lack of research on the topics mentioned above also caused some limitations for the current study. Decisions about care pathways and materials have been based on usual care in four Dutch hospitals and on the available literature, but information on subjects such as physiotherapy, OHRQoL, and recovery of treatment is limited. Therefore, we had to rely on expert opinions on these issues. Another limitation concerns the type of condylar fracture. As mentioned earlier, fractures of the head, as well as neck, and base of the condyle can be treated either by surgical, conservative or expectative treatment. Bilateral condylar fractures or concomitant facial fractures however have not been taken into account, although these represent a majority of the cases [10], due to the difficulties this would cause in comparing costs.

Another limitation is that we only partially distinguish between different types of condylar fractures and associated surgical or conservative treatment choices (e.g., the number of plates or brackets needed, the type of plate or brackets used) because we could not reliably obtain this information. Therefore, we included options that are usual care in the Dutch hospitals. Both cheap and more expensive options have been included, to avoid an under- or overestimation of costs.

Finally, due to the lack of strict treatment protocols, it is difficult to extrapolate the costs of treating condylar fractures to the whole of the Netherlands, let alone other countries. Although the Dutch health care system may be comparable to those in other Western European countries, it is very different from the US system. However, even if care pathways or materials vary between settings, the considerable differences in costs between the different treatment options will probably remain, which means that the same pattern is expected in other settings.

## 5 Conclusion

There is a considerable difference in actual costs between surgical, conservative and expectative treatment of condylar fractures. Direct treatment costs of surgical treatment are three to five times higher than those of conservative treatment. However, indirectly related costs due to the recovery weigh heavily on this balance, but also when comparing total costs the surgical care path is a factor 1.5 more expensive than that of either conservative or expectative treatment. Recovery from surgery would need to be around two weeks shorter than recovery from either conservative or expectative treatment, for the surgical treatment to be cost-effective.

## Statement of clinical relevance

The different treatments for condylar fractures have comparable outcomes, but surgical treatment costs are much higher than those of conservative treatment. This cost-of-illness study is a first step towards implementing value-based healthcare for condylar fractures. (Rozeboom et al., 2017 (2) https://doi.org/10.1016/J.IJOM.2016.11.009, https://doi.org/10.1016/J.IJOM.2017.06.018).

## Author contribution statement

Loreine Helmer: Conceived and designed the experiments; Performed the experiments; Analyzed and interpreted the data; Wrote the paper. Leander Dubois: Frank Lobbezoo: Jan de Lange: Contributed reagents, materials, analysis tools or data; Wrote the paper. Judith Bosmans: Conceived and designed the experiments; Analyzed and interpreted the data; Wrote the paper.

## Data availability statement

Data included in article/supp. Material/referenced in article.

## Declaration of competing interest

The authors declare that they have no known competing financial interests or personal relationships that could have appeared to influence the work reported in this paper.
