# Supplementary material for: Healthcare costs of different treatment options for condylar fractures
Source: Heliyon. 2023 Sep 9;9(9):e19851. doi: 10.1016/j.heliyon.2023.e19851 (PMC10559232; doi:10.1016/j.heliyon.2023.e19851)
Supplement: Multimedia component 1 [file mmc1.docx]

## Supplementary information, specification of other phases of the care pathways

Table S1. Intake and diagnostics

| Care pathway | Costs minimal | Costs maximal | Source |
| --- | --- | --- | --- |
|  |  |  |  |
| **Costs intake & diagnostics** |  |  |  |
| Presentation emergency room & consultation medical specialist | 259 |  | Richtlijn Zorginstituut^9^ |
| Computed tomography (CT) or Cone-beam computed tomography (CBCT), brain image | 129 |  | Richtlijn Zorginstituut^9^ |
|  |  |  |  |
| ***Total costs intake & diagnostics for all treatment options*** | **388** |  |  |
| *Part human resources* | 259 |  |  |
| *Part materials & diagnostics* | 129 |  |  |

Table S2. Follow-up appointments: overview per treatment

| Care pathway | Details | Costs minimal | Costs maximal | Source |
| --- | --- | --- | --- | --- |
|  |  |  |  |  |
| **Costs follow-up surgical treatment** |  |  |  |  |
| Computed tomography (CT) or Cone-beam computed tomography (CBCT), brain image | | 129 |  | Richtlijn Zorginstituut^9^ |
| Follow-up appointments 7x | Outpatient time clinic: weighed average 91 €/10min | 637 |  | Richtlijn Zorginstituut^9^ |
| Physiotherapy appointments 8x | Physiotherapy per appointment €33 | 264 |  | Richtlijn Zorginstituut^9^ |
| Orthopantomogram (OPG) |  | 50 |  | Bazargani et al, 2013^26^ |
|  |  |  |  |  |
| ***Total costs follow-up surgical treatment*** |  | **1080** |  |  |
| *Human resources* |  | 901 |  |  |
| *Materials & diagnostics* |  | 179 |  |  |
|  |  |  |  |  |
| **Costs follow-up conservative treatment** |  |  |  |  |
| CT/CBCT (brain image) |  | 129 |  | Richtlijn Zorginstituut^9^ |
| Follow-up appointments 7x | Outpatient time clinic: weighed average 91 €/10min | 637 |  | Richtlijn Zorginstituut^9^ |
| Physiotherapy appointments 8x | Physiotherapy per appointment €33 | 264 |  | Richtlijn Zorginstituut^9^ |
| OPG |  | 50 |  | Bazargani et al, 2013^26^ |
|  |  |  |  |  |
| ***Total costs follow-up conservative treatment*** |  | **1080** |  |  |
| *Part human resources* |  | 901 |  |  |
| *Part materials & diagnostics* |  | 179 |  |  |
|  |  |  |  |  |
| **Costs follow-up expectative treatment** |  |  |  |  |
| CT/CBCT (brain image) |  | 129 |  | Richtlijn Zorginstituut^9^ |
| Follow-up appointments 7x | Outpatient time clinic: weighed average 91 €/10min | 637 |  | Richtlijn Zorginstituut^9^ |
| Physiotherapy appointments 8x | Physiotherapy per appointment €33 | 264 |  | Richtlijn Zorginstituut^9^ |
| OPG |  | 50 |  | Bazargani et al, 2013^26^ |
|  |  |  |  |  |
| ***Total costs follow-up expectative treatment*** |  | **1080** |  |  |
| *Human resources* |  | 901 |  |  |
| *Materials & diagnostics* |  | 179 |  |  |

Table S3. Recovery: different scenarios and total costs per treatment for each scenario

| Care pathway | Details | Base case** | Scenario A** | Scenario B** | Source |
| --- | --- | --- | --- | --- | --- |
|  |  |  |  |  |  |
| **Costs recovery surgical treatment** |  |  |  |  |  |
| Sick leave | Sick leave costs per hour: 34.75* 8 hours = 278 €/day | 14 | 7 | 21 | Richtlijn Zorginstituut^9^ |
| Caretaker cancelled job |  |  |  |  |  |
|  |  |  |  |  |  |
| ***Total costs recovery surgical treatment*** |  | **3892** | **1946** | **5838** |  |
| *Human resources* |  | 3892 | 1946 | 5838 |  |
| *Materials & diagnostics* |  | 0 | 0 | 0 |  |
|  |  |  |  |  |  |
| **Costs recovery conservative treatment** |  |  |  |  |  |
| Sick leave | Sick leave costs per hour: 34.75* 8 hours = 278 €/day | 14 | 21 | 7 | Richtlijn Zorginstituut^9^ |
| Caretaker cancelled job |  |  |  |  |  |
|  |  |  |  |  |  |
| ***Total costs recovery conservative treatment*** |  | **3892** | **5838** | **1946** |  |
| *Human resources* |  | 3892 | 5838 | 1946 |  |
| *Materials & diagnostics* |  | 0 | 0 | 0 |  |
|  |  |  |  |  |  |
| **Costs recovery expectative treatment** |  |  |  |  |  |
| Sick leave | Sick leave costs per hour: 34.75* 8 hours = 278 €/day | 14 | 21 | 7 | Richtlijn Zorginstituut^9^ |
| Caretaker cancelled job |  |  |  |  |  |
|  |  |  |  |  |  |
| ***Total costs recovery expectative treatment*** |  | **3892** | **5838** | **1946** |  |
| *Human resources* |  | 3892 | 5838 | 1946 |  |
| *Materials & diagnostics* |  | 0 | 0 | 0 |  |
|  |  |  |  |  |  |
| **Total costs entire care pathway surgical*** |  | **9012.1** | **7066.1** | **10958.1** |  |
| **Total costs entire care pathway conservative*** |  | **6424.9** | **8370.9** | **4478.9** |  |
| **Total costs entire care pathway expectative*** |  | **5434.7** | **7380.7** | **3488.7** |  |

**For this sum, the less expensive/'best' option was elected*

***Three different scenarios of the recovery period after treatment of a condylar fracture. The base case is used in our study, with 14 days of sick leave for every treatment option. Scenario A includes seven days of sick leave after surgical treatment and 21 days of sick leave after conservative and expectative treatment. Scenario B consists of 21 days of sick leave after surgical treatment and seven days of sick leave after conservative and expectative treatment.*
